# Optic nerve sheath diameter and its association with brain swelling in pediatric cerebral malaria: a retrospective study

**DOI:** 10.3389/fped.2024.1295254

**Published:** 2024-02-15

**Authors:** Madiha Q. Raees, Montfort Benard Gushu, Terrie E. Taylor, Karl B. Seydel, Hunter J. Wynkoop, Nicole F. O’Brien

**Affiliations:** ^1^ Division of Critical Care, Department of Anesthesiology and Critical Care, Children’s Hospital of Philadelphia, Philadelphia, PA, United States; ^2^ Blantyre Malaria Project, Kamuzu University of Health Sciences, Blantyre, Malawi; ^3^ Department of Osteopathic Medical Specialties, College of Osteopathic Medicine, Michigan State University, East Lansing, MI, United States; ^4^ Division of Critical Care Medicine, Department of Pediatrics, Nationwide Children’s Hospital, The Ohio State University, Columbus, OH, United States

**Keywords:** pediatrics, Africa South of the Sahara, cerebral malaria, falciparum malaria, brain edema, point-of-care technology

## Abstract

**Introduction:**

Mortality in pediatric cerebral malaria (CM) in low- and middle-income countries (LMICs) is associated with brain swelling on magnetic resonance imaging (MRI); however, MRI is unavailable in most LMICs. Optic nerve sheath diameter (ONSD) measurement is an inexpensive method of detecting increased intracranial pressure compared with the invasive opening pressure (OP). Our primary objective was to determine if increased ONSD correlated with brain swelling on MRI in pediatric CM. Our secondary objective was to determine if increased ONSD correlated with increased OP and/or poor neurological outcome in pediatric CM. We hypothesized that increased ONSD would correlate with brain swelling on MRI and increased OP and that ONSD would be higher in survivors with sequelae and non-survivors.

**Methods:**

We performed a retrospective chart review of children aged 0–12 years in Blantyre, Malawi, from 2013 to 2022 with CM as defined by the World Health Organization. Brain swelling on admission MRI was characterized by brain volume scores (BVS); severe swelling was scored as 7–8, mild-to-moderate as 4–6, normal as 3. The admission ONSD was measured via ultrasound; it was defined as abnormal if it was >4.5 mm in children >1 year and >4 mm in children <1 year. Favorable outcome was defined as a normal neurological exam on discharge in survivors. The primary and secondary objectives were evaluated using Spearman's correlation; and the demographics were compared using chi-square and the Kruskal–Wallis test (Stata, College Station, TX, USA).

**Results:**

Median age of the 207-patients cohort was 50 months [interquartile range (IQR) 35–75]; 49% (*n* = 102) were female. Of those, 73% (*n* = 152) had a favorable outcome, and 14% (*n* = 30) died. Twenty-nine (14%) had a normal BVS, 134 (65%) had mild-to-moderate swelling, and 44 (21%) had severe swelling. ONSD was elevated in 86% (*n* = 178) of patients, while 12% of patients had increased OP. There was a weakly positive correlation between BVS and ONSD (*r* = 0.14, *p* = 0.05). The median ONSD was not significantly different compared by discharge outcome (*p* = 0.11) or by BVS (*p* = 0.18).

**Conclusion:**

ONSD was not a reliable tool to correlate with BVS, neurological outcome, or OP in children with CM. Future studies to identify alternative methods of early identification of CM patients at highest risk for morbidity and mortality are urgently needed.

## Introduction

Malaria is a parasite-borne disease caused by the protozoan *Plasmodium*, transmitted to humans through the saliva of *Anopheles* mosquitos. The most severe form of the disease is caused by *Plasmodium falciparum* and is estimated to cause hundreds of thousands of deaths annually, the majority of which occur in sub-Saharan Africa. Children are a particularly vulnerable population due to a lack of acquired immunity, and are more susceptible to developing severe malarial anemia and cerebral malaria (CM), the most severe form of falciparum malaria (1, 2).

In CM, the most likely mechanism of morbidity and mortality is due to the progressively increasing brain volume that leads to intracranial hypertension, cerebral herniation, and ultimately death. Erythrocytes infected with *P. falciparum* lose their usual deformability and have increased cytoadherence to vascular endothelial cells, leading to sequestration and microvascular occlusion throughout the systemic circulation (3). An association has been demonstrated between increased brain volume on magnetic resonance imaging (MRI) and fatal outcomes from CM in Malawian children (4). Unfortunately, most hospitals in malaria-endemic regions do not have access to MRI, therefore proving impractical for most local practitioners. In the search for more cost-effective, readily available alternatives, optic nerve sheath diameter (ONSD) measurement, a point-of-care method to detect increased intracranial pressure (ICP) non-invasively in pediatrics, may be helpful (5–13).

Optic nerve sheath diameter utilizes a low-cost, well-established, and widely available technology. The optic nerve is readily visualized using a linear ultrasound probe applied to a closed eyelid in the transverse view (5). In pediatrics, increased ONSD has been associated with increased ICP in ventriculoperitoneal shunt failure, idiopathic intracranial hypertension, diabetic ketoacidosis, and traumatic brain injury (6–9). The published results of ONSD measurement in CM have inconsistently demonstrated an association with depth of coma and/or long-term neurological outcome (13–16). We developed our primary objective of determining if abnormally elevated admission ONSD correlates with an increased brain volume score (BVS) on MRI and our secondary objectives of examining if elevated ONSD correlates with an elevated opening pressure (OP) on lumbar puncture and a poor outcome at hospital discharge. We hypothesized that those patients with increased ONSD on admission would have increased brain swelling on admission brain MRI, an increased OP, and/or would be more likely to have a new neurological deficit or die.

## Methods

This was a retrospective observational study performed via chart review of CM patients admitted to the pediatric research ward at the Queen Elizabeth Central Hospital in Blantyre, Malawi, over a period of 10 years. We analyzed children 6 months–12 years of age who presented with CM from 2013 to 2022. The inclusion criteria required physical and laboratory exam consistent with CM as defined by the World Health Organization [Blantyre coma score ≤2 (on a scale from 0 to 5 with lower scores demonstrating decreased consciousness); parasitemia with *P. falciparum*; with no other reasonable explanation for coma] (17). The exclusion criteria included age greater than 12 years and other comorbidities, such as trauma, suspected non-malarial infection, or pregnancy. Children with pre-existing developmental delay or neurological conditions were not excluded. Demographic data and pre-illness caregiver questionnaire, admission fundoscopy for malarial retinopathy, admission brain swelling as defined by the BVS on MRI, admission ONSD, and neurological outcomes at discharge were collected. ONSD measurement and brain MRI were obtained on the same day in a sequential fashion.

The brain volume score on MRI is a numerical value assigned by two radiologists blinded to the patients’ clinical status based on the appearance of the cerebral hemispheres, ranging from 1 to 8, and was part of the routine workup of CM patients admitted to the research ward. Discordant reads were resolved by the addition of a third neuroradiologist read to serve as consensus. A score of 1 indicated marked atrophy and a score of 2 was consistent with mild atrophy. A score of 3 was assigned to those with a normal brain volume. A score of 4 showed slightly increased volume, 5 showed mildly increased volume, 6 showed moderately increased volume, 7 showed substantially increased volume with diffuse sulcal and cisternal effacement without herniation, and 8 showed effacement of the sulci and cisterns with evidence of herniation (4). We further subdivided this as atrophy (BVS 1–2), normal (BVS 3), mild-to-moderate swelling (BVS 4–6), and severe swelling (BVS 7–8). From 2013 to 2019, MRI scans were performed on the 0.35-T Signa Ovation Excite MRI scanner (General Electric). From 2021 onward, MRI was performed on the 64 mT Hyperfine Swoop Portable MRI scanner.

**Figure 1:**
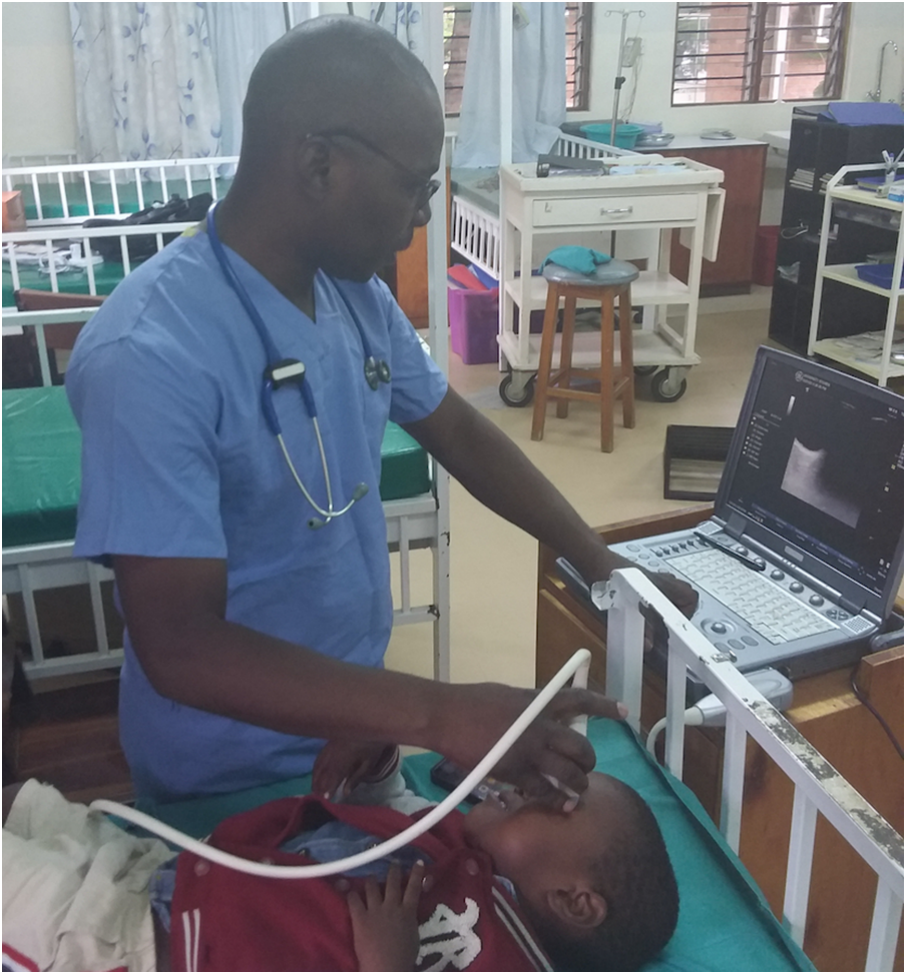
B-mode technique of bedside ultrasound operation to visualize optic nerve sheath and measure diameter.

**Figure 2:**
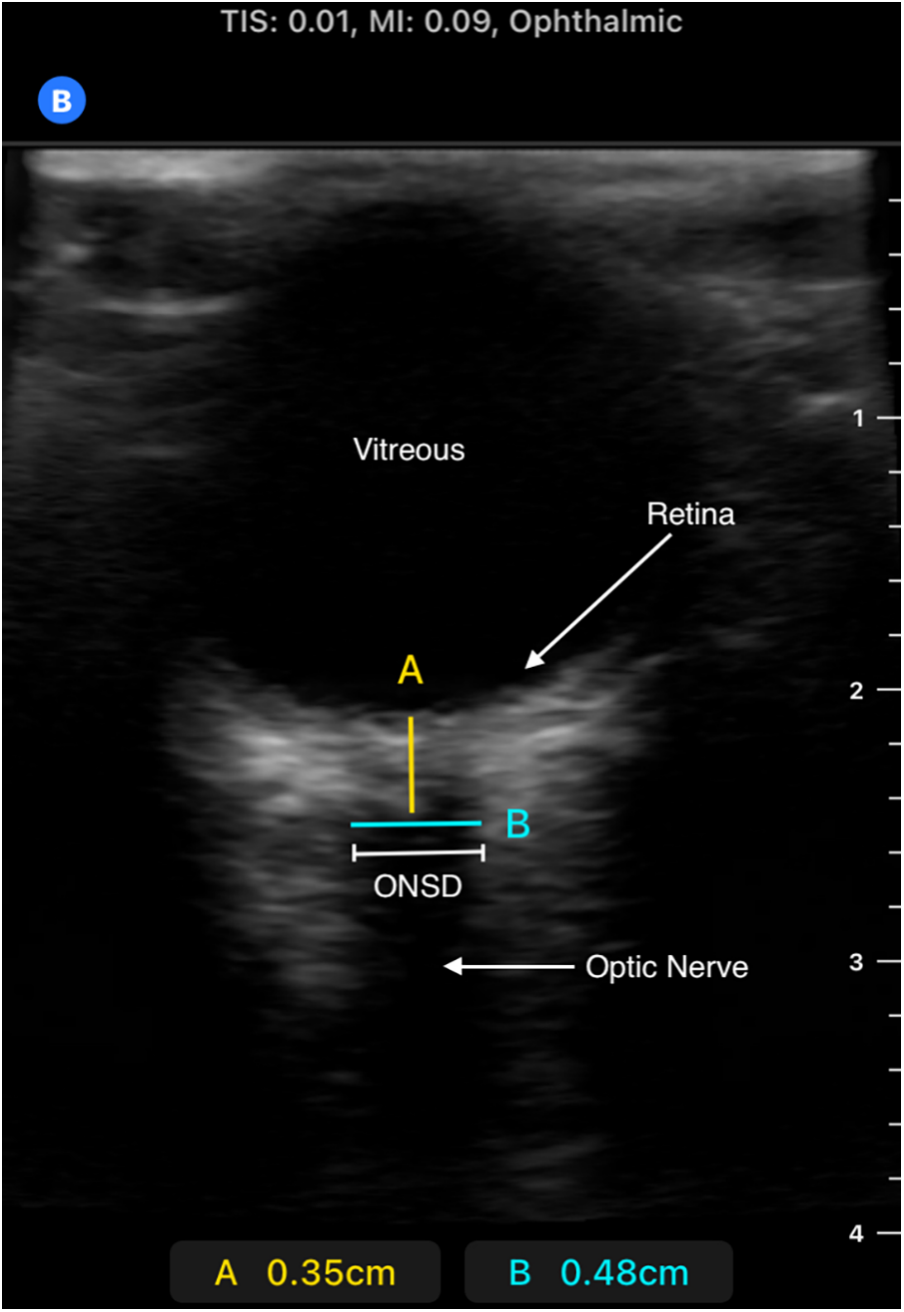
Ultrasound image of retina, optic nerve, and optic nerve sheath. Image obtained using B-mode technique; measurement made approximately 3 mm posterior to retina (measurement “A”). The anechoic “strip” in this region is the optic nerve. A hypoechoic sheath is visible immediately lateral to the optic nerve, representing the ideal location to take the ONSD measurement, represented by measurement “B”. Please note this is a representative image obtained from a pediatric patient outside of our study from a Butterfly IQ+ device which was not used in our patient population.

Optic nerve sheath was measured on admission via portable ultrasonography using the B-mode technique by one of three trained clinicians and was also part of the routine admission workup of CM patients admitted to the research ward (Figures 1, 2). A GE Logiq e portable ultrasound was utilized to obtain ONSD. This measurement was obtained shortly after the patient's arrival to the ward, along with the performance of a lumbar puncture for sample collection, and preceded the MRI; thus, clinicians obtaining ONSD measurements were blinded to the MRI findings. Both eyes had a thick layer of ultrasound gel placed over them and were maintained in a closed position. The transverse linear probe was then used to isolate the optic nerve using an anterior transbulbar approach. The sheath, containing dura, arachnoid, pia, and the nerve itself, was measured ∼3 mm posterior to the retina. The measurements were obtained bilaterally and a minimum of three values on each eye were averaged to obtain a single value for each patient. A score ≥4.5 mm in children >1 year and >4 mm in children <1 year was defined as abnormal (18). In patients who underwent lumbar puncture, OP was measured utilizing a manometer, with data collected in mm H_2_O; values greater than 270 mm H_2_O were defined as elevated, chosen based on previously published reference values (19). Favorable outcome in survivors was defined as a grossly normal neurological exam on discharge.

Spearman's correlation was used given the non-parametric data set to compare a continuous variable (ONSD) to an ordinal variable (BVS). Patients were also divided into groups based on BVS [normal, mild-to-moderate (BVS 4–6), and severe (7–8)] and median ONSD value and median OP value were compared using the Kruskal–Wallis test. Following this, patients were grouped by hospital discharge outcome (survivors, survivors with sequelae, and non-survivors) and median ONSD value and median OP value were compared using chi-square test. The statistics were completed using Stata (College Station, TX). Only univariable testing was performed. The parent study (NIH # 1U01AI126610, registered with clinicaltrials.gov NCT03300648) for which these data were collected was approved by the Michigan State University IRB (IRB# 16-876M) and the Malawi College of Medicine Research & Ethics Committee (P.09/16/2024); written informed consent was provide by parents or guardians prior to collection of any data.

## Results

Demographic, admission, and outcomes data can be found in Table 1. For the 207 patients, the median age was 50 months [interquartile range (IQR) 35–75] with an even gender split (49% females, *n* = 102). More than three quarters (79%, *n* = 164) of our cohort had malarial retinopathy, a characteristic finding consisting of retinal whitening, vessel changes, and retinal hemorrhages seen in most children with cerebral malaria (20). Almost three quarters (73%, *n* = 152) of the patients had a history of seizures at some point since onset of illness. Approximately 86% (*n* = 178) had brain swelling on MRI—65% with mild-to-moderate, while 21% had severe. ONSD was elevated in 86% (*n* = 178) of patients, while opening pressure via lumbar puncture was elevated in 12% in those on whom it was performed (*n* = 19/157). Most patients (73%) survived without grossly abnormal sequelae. Similar proportions of patients died and had gross neurological abnormalities on discharge. As is consistent with our previously published data, there was a positive correlation between BVS and poor neurological outcome (*r* = 0.274, *p* < 0.01).

**Table 1:** Patient demographics, admissions, and outcomes data.

	*N* = 207
Age (months), median [IQR (25th–75th)]	50 (35–75)
Female, *n* (%)	102 (49)
Retinopathy positive, *n* (%)	164 (79)
Seizures prior to admission, *n* (%)	152 (73)
Elevated opening pressure on LP, *n* (%), *n* = 157	19 (12)
Brain volume score, *n* (%)	
Normal (BVS 3)	29 (14)
Mild-to-moderate swelling (BVS 4–6)	134 (65)
Severe swelling (BVS 7–8)	44 (21)
Elevated ONSD, *n* (%)	178 (86)
Discharge outcome, *n* (%)	
Survived without sequelae	152 (73)
Survived with sequelae	25 (12)
Died	30 (15)

This table contains information on patients’ demographic and historical data and details of diagnostic workup along with discharge outcome. Variable sample sizes are the result of missing data.

**Figure 3:**
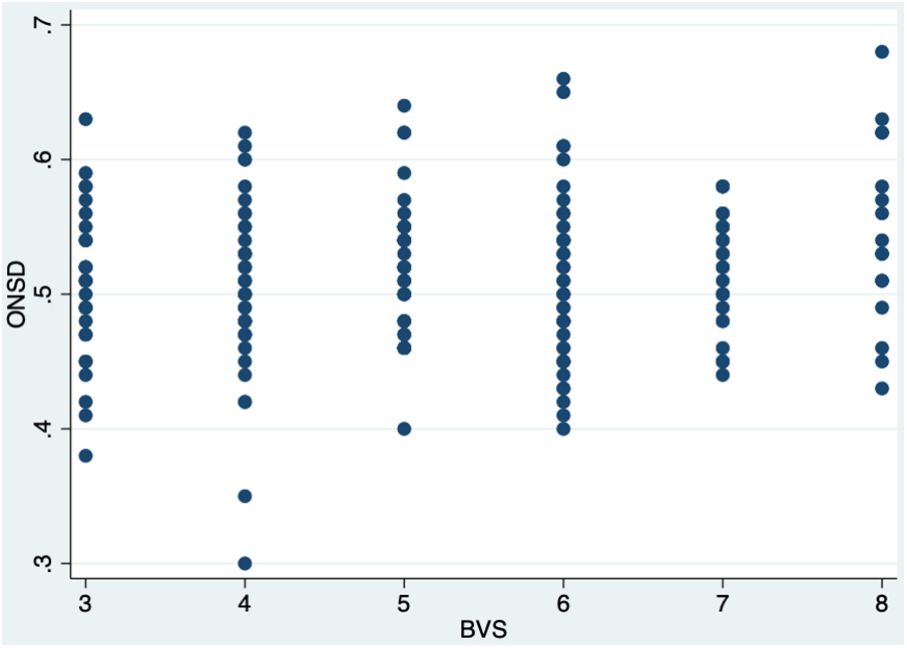
Scatter plot comparing BVS values (on x-axis) and ONSD in centimeters (on y-axis).

When stratified by BVS group, ONSD was elevated in 79% (*n* = 23) of the normal group, 87% (*n* = 117) of the mild-to-moderate group, and 86% (*n* = 38) of the severe group; this was not statistically significant between groups (*p* = 0.79). Opening pressure was elevated in 4% (*n* = 1/24) of normal, 13% (*n* = 12/95) of mild-to-moderate, and 16% (*n* = 6/38) of the severely affected patients; this was also not statistically significant between groups (*p* = 0.22). There was a weakly positive correlation between BVS and ONSD (*r* = 0.14, *p* = 0.05, Figure 3) and but not between ONSD and cerebrospinal fluid (CSF) opening pressure(*r* = 0.138, *p* = 0.085). Median ONSD and median OP were not significantly different between BVS groups (*p* = 0.18 and *p* = 0.18) as shown in Table 2. Median ONSD was not significantly different between discharge outcome group (*p* = 0.11), nor was median OP (*p* = 0.10) as is seen in Table 3. Median ONSD was not significantly different between age groups most likely to have a fontanelle (26 months or younger) compared with those who would not (older than 26 months, *p* = 0.38), nor was it significantly different between those who had a history of seizure prior to presentation compared with those who had not (*p* = 0.749).

**Table 2:** Optic nerve sheath diameter and opening pressure by brain volume score group.

	Normal BVS (3), *n* = 29	Mild-to-moderate swelling (BVS 4–6), *n* = 134	Severe swelling (BVS 7–8), *n* = 44	*p*-value
Median ONSD in mm (IQR)	5.1 (4.7–5.4)	5.1 (4.7–5.4)	5.3 (4.9–5.6)	0.18
Median OP in mm H_2_O (IQR)	157.5 (135–200)	170 (125–220)	185 (140–260)	0.18

This table compares the median ONSD and median OP among each of these groups (normal, mild-to-moderate, and severe BVS).

**Table 3:** Optic nerve sheath diameter and opening pressure by discharge outcome.

	Survivors, *n* = 152	Survivors with sequelae, *n* = 25	Non-survivors, *n* = 30	*p*-value
Median ONSD in mm (IQR)	5.2 (4.8–5.5)	4.9 (4.5–5.4)	5.15 (4.8–5.7)	0.11
Median OP in mm H_2_O (IQR)	170 (130–220)	150 (120–240)	190 (170–230)	0.10

This table compares the median ONSD and median OP among each of these groups (survivors, survivors with sequelae, non-survivors).

## Discussion

Cerebral malaria remains an important cause of morbidity and mortality in children. Autopsy studies of children with CM demonstrate sequestration of parasites in cerebral capillaries. In addition, there is frequently evidence of gross brain swelling with associated necrosis (21). This is most consistent with parasite-induced erythrocyte obstruction of the cerebral microvasculature leading to cerebral edema, herniation, and ultimately death in these patients. Our work in CM in Malawian children has demonstrated that those with increased brain swelling on MRI at presentation are at highest risk of death (4); this finding was reproduced in our patient cohort as well.

In regions with limitations in access to medical diagnostics and therapeutics, such as many of those where malaria is endemic, the ability to risk stratify these patients would be incredibly valuable. While the association of the MRI findings of these patients with outcomes has allowed for strides to be made in identifying the pathophysiology of CM, it is not a practical prognostic tool to implement in malaria-endemic regions subject to thoughtful distribution of limited resources. Thus, a low-cost, non-invasive tool that can serve as a surrogate for MRI has been sought; ONSD measurement has been suggested as this alternative.

The optic nerve is a direct connection to the central nervous system that can be easily visualized by even novice medical practitioners. The optic nerve sits in a sheath that contains the optic dura, pia, and subarachnoid space. The optic subarachnoid space is anatomically contiguous with the intracranial subarachnoid space—hence, marked increases in the intracranial pressure would be transmitted to the optic subarachnoid space, increasing the amount of fluid around the optic nerve and therefore increasing the ONSD (7). ONSD is increased in cases of ventriculoperitoneal shunt malfunction, diabetic ketoacidosis, and acute liver failure presumed secondary to increased intracranial pressure (5, 7, 8).

ONSD measurement in our CM patients did not correlate with brain volume scores based on brain swelling on MRI, nor did it correlate with outcome. Furthermore, the lack of correlation with increased opening pressure as measured by manometry during lumbar puncture in these patients suggests that the ONSD does not accurately reflect ICP in our patients. In our population, nearly all patients had elevated ONSD per published norms, even with up to 15% (*n* = 32) of children possibly having an open fontanelle to serve as a “pop off” for increased pressure. Notably, ONSD findings in this cohort are markedly different from a previously studied group of Malawian children with CM in Blantyre by Beare et al. in 2012; in the study by Beare et al., ONSD enlargement was less prevalent than increased opening pressure via lumbar puncture (49% vs. 95%) and neurological sequelae were more commonly seen in children with increased ONSD (16). This study had a smaller patient population, which may have contributed to the conclusions drawn. In addition, markedly different cutoffs were used to define increased opening pressure via lumbar puncture. Most notably, however, the median opening pressure obtained on lumbar puncture in those with an elevated ONSD is strikingly lower in our study when compared with that of Beare et al. (170 vs. 220 mm), reflecting a decrease in the prevalence of increased ICP on admission in our patient population.

One possible explanation for our findings could be that the increased intracranial pressure occurs in an intermittent or “spike” fashion as it does in disease states such as traumatic brain injury; a study performed in Kenyan children with CM showed an episodic nature to ICP elevations as captured by an invasive monitor (22). In that case, it is possible that at the time of admission, ONSD was persistently elevated, reflecting a previous “spike” in ICP (e.g., brief elevation due to seizure or fever), even if the ICP had since normalized, resulting in a normal opening pressure via lumbar puncture. The persistence of ONSD expansion beyond resolution of increased ICP has been demonstrated in traumatic brain injury in the adult population, limiting ONSD's use in the acute phase (23). In addition, only one method of measuring ONSD was employed in this study that has limitations in its imaging ability of small structures, an example of which is the optic nerve (24). It is possible that with the introduction of a second, possibly more accurate technique, such as A-scan ultrasonography, the prevalence of increased ONSD would be lower in our patient population.

This study has several important limitations. Despite being a large sample size compared with other studies of CM, our sample size remains relatively small when compared with larger studies completed in adults or in more common disease states, limiting statistical power. Multivariable testing was not completed. In addition, single ONSD measurement inclusion on admission without further measurements to correlate with changes in exam and serial MRI results also limit our results. Multiple operators of the ONSD ultrasound measurement were included in this study, leading to possible differences in technique and lack of standardization contributing to results. Furthermore, the particular ONSD technique described in this study, while the most commonly replicated in point-of-care studies due to its ease in performance with the most simple equipment setup, has innate limitations, including the inability to assess gaze direction because of closed eyelids and potential artifact from the choice of probe and sensitivity settings, as is evidenced by the wide range of suggested reference values (23).

## Conclusions

ONSD as measured by the B-scan technique was not a reliable tool to identify increased brain swelling as detected on MRI in children with CM, nor did it correlate with OP findings or hospital discharge outcomes. Future studies utilizing point-of-care ultrasound may find closer associations between MRI findings and ONSD with the use of more accurate techniques. Further work to identify an alternative method of early identification of children with CM that are at high risk for morbidity and mortality is urgently needed.

## Data Availability

The raw data supporting the conclusions of this article will be made available by the authors, without undue reservation.

## References

[1] Idro R Jenkins NE Newton CR. Pathogenesis, clinical features, and neurological outcome of cerebral malaria. Lancet Neurol. (2005) 4(12):827–40. 10.1016/S1474-4422(05)70247-7 16297841

[2] World Health Organization. Malaria. (nd). Available online at: https://www.who.int/news-room/fact-sheets/detail/malaria (accessed June 5, 2023).

[3] Idro R Marsh K John CC Newton CRJ. Cerebral malaria: mechanisms of brain injury and strategies for improved neurocognitive outcome. Pediatr Res. (2010) 68(4):267–74. 10.1203/PDR.0b013e3181eee738 20606600 PMC3056312

[4] Seydel KB Kampondeni SD Valim C Potchen MJ Milner DA Muwalo FW Brain swelling and death in children with cerebral malaria. N Engl J Med. (2015) 372(12):1126–37. 10.1056/NEJMoa1400116 25785970 PMC4450675

[5] Cannata G Pezzato S Esposito S Moscatelli A. Optic nerve sheath diameter ultrasound: a non-invasive approach to evaluate increased intracranial pressure in critically ill pediatric patients. Diagnostics. (2022) 12(3):767. 10.3390/diagnostics12030767 35328319 PMC8946972

[6] Young AMH Guilfoyle MR Donnelly J Scoffings D Fernandes H Garnett M Correlating optic nerve sheath diameter with opening intracranial pressure in pediatric traumatic brain injury. Pediatr Res. (2017) 81(3):443–7. 10.1038/pr.2016.165 27513519

[7] Kerscher SR Schöni D Hurth H Neunhoeffer F Haas-Lude K Wolff M The relation of optic nerve sheath diameter (ONSD) and intracranial pressure (ICP) in pediatric neurosurgery practice—part I: correlations, age-dependency and cut-off values. Childs Nerv Syst. (2020) 36(1):99–106. 10.1007/s00381-019-04266-1 31256241

[8] Vijay P Lal BB Sood V Khanna R Patidar Y Alam S. Dynamic optic nerve sheath diameter (ONSD) guided management of raised intracranial pressure in pediatric acute liver failure. Hepatol Int. (2021) 15(2):502–9. 10.1007/s12072-021-10139-0 33625660

[9] Tessaro MO Friedman N Al-Sani F Gauthey M Maguire B Davis A. Pediatric point-of-care ultrasound of optic disc elevation for increased intracranial pressure: a pilot study. Am J Emerg Med. (2021) 49:18–23. 10.1016/j.ajem.2021.05.051 34051397

[10] Kayadibi Y. Correlation between optic nerve sheath diameter (ONSD) and Rotterdam computer tomography scoring in pediatric brain injury. Ulus Travma Acil Cerrahi Derg. (2019) 26(2):212–21. 10.14744/tjtes.2019.94994 32185780

[11] Amakhian AO Obi-Egbedi-Ejakpovi EB Morgan E Adeyekun AA Abubakar MM. Correlation between optic nerve sheath diameter at initial head CT and the Rotterdam CT score. Cureus. (2023) 15(7):e41995. 10.7759/cureus.41995 37593265 PMC10428083

[12] Salahuddin N Mohamed A Alharbi N Ansari H Zaza KJ Marashly Q The incidence of increased ICP in ICU patients with non-traumatic coma as diagnosed by ONSD and CT: a prospective cohort study. BMC Anesthesiol. (2016) 16(1):106. 10.1186/s12871-016-0267-1 27776491 PMC5078918

[13] Chelly J Deye N Guichard J-P Vodovar D Vong L Jochmans S The optic nerve sheath diameter as a useful tool for early prediction of outcome after cardiac arrest: a prospective pilot study. Resuscitation. (2016) 103:7–13. 10.1016/j.resuscitation.2016.03.006 26995663

[14] Savi De Tové K-M De Tové Sissinto YS Adedemy DJ Akanni D Kiki M Yèkpè-Ahouansou P Sonographic measurement of optic nerve sheath diameter: a prognostic tool for childhood cerebral malaria? OJRad. (2019) 09(01):69–81. 10.4236/ojrad.2019.91007

[15] Murphy S Cserti-Gazdewich C Dhabangi A Musoke C Nabukeera-Barungi N Ultrasound findings in *Plasmodium falciparum* malaria: a pilot study. Pediatric Critical Care Medicine. (2011) 12(2):e58–63. 10.1097/PCC.0b013e3181e89992 20581730

[16] Beare NAV Glover SJ Lewallen S Taylor TE Harding SP Molyneux ME. Prevalence of raised intracranial pressure in cerebral malaria detected by optic nerve sheath ultrasound. Am J Trop Med Hyg. (2012) 87(6):985–8. 10.4269/ajtmh.2012.11-0459 23033398 PMC3516101

[17] Severe falciparum malaria. World Health Organization, communicable diseases cluster. Trans R Soc Trop Med Hyg. (2000) 94(Suppl 1):S1–90. PMID: 11103309

[18] Ballantyne J Hollman AS Hamilton R Bradnam MS Carachi R Young DG Transorbital optic nerve sheath ultrasonography in normal children. Clin Radiol. (1999) 54(11):740–2. 10.1016/S0009-9260(99)91176-5 10580764

[19] Avery RA Shah SS Licht DJ Seiden JA Huh JW Boswinkel J Reference range for cerebrospinal fluid opening pressure in children. N Engl J Med. (2010) 363(9):891–3. 10.1056/nejmc1004957 20818852 PMC3746184

[20] Beare NAV Taylor TE Harding SP Lewallen S Molyneux M. Malarial retinopathy: a newly established diagnostic sign in severe malaria. Am J Trop Med Hyg. (2006) 75(5):790–7. 10.4269/ajtmh.2006.75.790 17123967 PMC2367432

[21] Milner DA Whitten RO Kamiza S Carr R Liomba G Dzamalala C The systemic pathology of cerebral malaria in African children. Front Cell Infect Microbiol. (2014) 4(104):1–13. 10.3389/fcimb.2014.00104 25191643 PMC4139913

[22] Newton CR Crawley J Sowumni A Waruiru C Mwangi I English M Intracranial hypertension in Africans with cerebral malaria. Arch Dis Child. (1997) 76(3):219–26. 10.1136/adc.76.3.219 9135262 PMC1717090

[23] Kavi T Gupta A Hunter K Schreiber C Shaikh H Turtz AR. Optic nerve sheath diameter assessment in patients with intracranial pressure monitoring. Cureus. (2018) 10(11):e3546. 10.7759/cureus.3546 30648078 PMC6324852

[24] Abbinante G Vitiello L Coppola A Salerno G Gagliardi V Pellegrino A. Optic nerve ultrasound evaluation in children: a review. Diagnostics. (2023) 13(3):535. 10.3390/diagnostics13030535 36766639 PMC9914511

